# The Effect of the Flint Water Crisis Secondary to Increased Lead Levels in Drinking Water on Constipation in Children in the City of Flint, Michigan, USA

**DOI:** 10.7759/cureus.44189

**Published:** 2023-08-27

**Authors:** Khalid Al-Kharraz, Mohammad J Tabbah, Jenny LaChance, Jamal Kriem

**Affiliations:** 1 Pediatrics, Hurley Medical Center, Flint, USA; 2 Pediatric Gastroenterology, Hurley Medical Center, Flint, USA; 3 Public Health, Hurley Medical Center, Flint, USA; 4 Pediatric Gastroenterology, Beaumont Hospital, Royal Oak, USA

**Keywords:** abdominal pain, straining, water, lead, flint, children, constipation

## Abstract

Introduction

Constipation is a common condition in children, affecting almost one-third of the population at some point in childhood across the world. Functional constipation is the most common cause, with no clear etiology. From April 25, 2014, through October 16, 2015, the water source for the city of Flint residents was untreated Flint River water, which resulted in lead-contaminated drinking water. Lead poisoning has been associated with constipation and has multisystem sequelae, including neurological, muscular, and hematological impacts. Children may be especially vulnerable to this with their higher water intake-to-body weight ratio. There has been no previous study examining the possible relationship between the Flint water crisis and constipation in children. In our study, we aimed to see if the increased lead level in the water had any effect on constipation in children in Flint.

Methods

We included all children seen and diagnosed with constipation at Hurley Medical Center’s Pediatric Gastrointestinal (GI) Clinic. We included only children seen in 2013 (pre-water crisis) and 2017 (post-water crisis). Children with chronic neurologic disorders, celiac disease, hypothyroidism, diabetes, Hirschsprung’s disease, short bowel syndrome, and gastrointestinal surgeries were excluded. We looked at the age of presentation, associated symptoms, medications used, need for hospital admission or emergency department (ED) visits, and improvements at follow-up.

Results

A total of 79 patients were included in the study. There were 29 patients from 2013 and 50 patients from 2017 (post-lead exposure period). The rate of constipation referrals to the GI clinic for the Flint population of children was significantly higher in 2017 (p=0.001). The most common associated symptom was abdominal pain in both groups. Straining was more prominent in the 2017 group (60%) compared to the 2013 group (34.5%, p=0.029). There was no clinical or statistically significant difference between the groups noted in abdominal pain, blood in the stool, fecal incontinence, vomiting, history of urinary tract infection (UTI), abdominal distention, or stool impaction.

Conclusions

The number of patients referred to Hurley’s Pediatric GI Clinic for constipation increased after the lead water crisis in Flint. Moreover, straining has significantly increased in post-lead exposure compared to pre-lead exposure. There was no clinical or statistically significant difference noted in abdominal pain, blood in the stool, fecal incontinence, vomiting, history of UTI, abdominal distention, or stool impaction between both groups. A larger study would need to be done to confirm these findings, rule out other cofactors, and look into minerals in water and their effect on intestine innervations.

## Introduction

Constipation is a common presentation in the pediatric age group, affecting up to one-third of children [1]. Constipation, defined as per Rome IV criteria, is two or more of the following symptoms: two or fewer defecations in the toilet per week in a child of a developmental age of at least four years; at least one episode of fecal incontinence per week; history of retentive posturing or excessive volitional stool retention; history of painful or hard bowel movements; presence of a large fecal mass in the rectum; a history of large diameter stools that can obstruct the toilet; or, after appropriate evaluation, the symptoms cannot be fully explained by another medical condition [2].

The most common etiology is functional constipation in 96% of the patients, and the rest are organic causes, including Hirschsprung’s disease, hypothyroidism, hypercalcemia, spinal lesions, and medication side effects [3].

Constipation is more common in children in the two- to four-year-old age group when they start toilet training. No clear cause led to functional constipation; however, genetics might play a factor in it [4].

Stimulant laxatives like polyethylene glycol are the safe and tolerated choice of treatment [5], along with behavior therapy including education and toilet training. There is not sufficient data to support the use of prebiotics, probiotics, and symbiotics in the treatment of children with functional constipation [6].

Water intake has been assessed in the literature for constipation. Studies suggest lower water intake is associated with a higher risk of constipation. More water intake might be beneficial for the prevention and treatment of mild constipation [7].

In 2016, the water lead crisis was noted in Flint. The lead crisis happened after changing the water supply in the city in April 2014. The lead level increased three times from its baseline in 2013. The incidence of elevated blood lead levels in children reached 4.9% in Flint [8].

There is no safe level of lead in the blood; however, the Centers for Disease Control and Prevention (CDC) currently uses the cutoff of 3.5 micrograms per deciliter to determine if it is within normal levels [9]. Lead poisoning in children can lead to brain damage, anemia, decreased renal function, muscle weakness, learning disabilities, attention deficit disorders, and impaired fine and gross motor skills [10].

There has been no previous study to examine the effect of the Flint water crisis on constipation in children. Lead has a negative impact on the neurological and muscular systems, and both might contribute to worsening constipation severity and prevalence. We are aiming to see if exposure to increased lead in water has any effect on the characteristics of constipation in children.

## Materials and methods

It was a retrospective cohort study. We included all children referred to Hurley Pediatric GI Clinic, Flint, USA, for constipation who were diagnosed with constipation at the end of the visit. We included children seen one year before the crisis and one year after the crisis. We chose the years 2013 and 2017 as the water supply was changed in mid-2014 and was completely fixed in 2016. We excluded children with chronic neurologic disorders, celiac disease, hypothyroidism, diabetes, Hirschsprung’s disease, children with short bowel syndrome, and children with gastrointestinal surgeries. We used the zip code of Flint to limit patients to those who live in the city and were exposed to the lead crisis.

We collected data using EPIC SlicerDicer using our inclusion and exclusion criteria. We reviewed patients’ charts from clinic visits and up to five years after their clinic visit. We collected the following data: demographic data: age (at visit), gender, and race; symptoms of nausea, vomiting, abdominal pain, straining, blood in the stool, fecal incontinence, abdominal distention, fecal impaction; complete blood count (CBC), electrolyte, and lead levels; imaging done including X-ray, CT scan, and barium enema; and endoscopies if found. Patient charts were reviewed for five years after the initial visit to see if they needed admissions or ED visits for clean-out and to see if the first treatment regimen showed improvement or not.

The chi-square analysis compared the differences in categorical variables before and after lead exposure. These categorical variables include gender, reported race, age group, hospital admission, ED intervention, presentation or symptoms (straining, blood in stool, etc.), blood workup (CBC, electrolyte, thyroid stimulating hormone (TSH), etc.), images taken, and management. An independent t-test was used to compare the mean age at presentation between the time periods.

## Results

From January 1, 2013, to December 31, 2013, 32 patients were referred to the GI clinic for constipation. From these 32, three patients were excluded, including one patient with myelomeningocele and spina bifida and two patients from the 48532 zip code. Patients from the 48532 zip code were excluded as it covers only a tiny area of the city of Flint, while the majority of the area was not involved in the lead crisis.

On the other hand, from January 1, 2017, to December 31, 2017, 66 patients were referred to our GI clinic after lead exposure, and 16 patients were excluded. Three of them had no constipation diagnosis linked to them: two patients had cerebral palsy, one had a traumatic brain injury, one had bowel surgery, and one was diagnosed with Hirschsprung’s disease. Eight patients were excluded from the 48532 zip code. A total of 79 patients were included: 29 patients before lead exposure and 50 patients post-lead exposure.

There were 65.5% female patients in the pre-lead exposure group and 64% in the post-lead exposure group. Males made up 34.5% and 36% of the pre-lead exposure group and post-lead exposure group, respectively. There was no significant statistical difference between genders. African Americans were the majority of the patients, with 65.5% and 56.3% in the pre-lead exposure group and post-lead exposure group, respectively. Of the patients, 29% and 42.9% identified as white [Table 1].

**Table 1:** Demography of patients included in the study

	Pre-Lead Exposure	Post-Lead Exposure	Total
Total Patients	29	50	79
Male	10 (34.5%)	18 (36%)	28 (35.4%)
Female	19 (65.5%)	32 (64%)	51 (64.6%)
African American	19	27	46 (58%)
White	8	20	28(35.4%)
Other (Latin, Arabs, etc.)	2	1	3(3.8%)
Mean age at presentation	5.9y	8.2y	N/A
1-12 months old	4 (13.7%)	7 (14%)	11 (13.9%)
13-36 months old	6 (20.7%)	0	6 (7.6%)
3-6 years old	7 (24%)	12 (24%)	19 (24%)
7-12 years old	9 (31%)	21 (42%)	30 (38%)
13-18 years old	3 (10 %)	10 (20%)	13 (16.5%)

The mean age of presentation at the clinic was 5.9 years before lead exposure and 8.2 years after lead exposure. The difference was statistically significant, with a P value of 0.04. The majority of patients who came to the clinic were from the seven- to twelve-year-old age group in both groups (31% and 42%, respectively).

Admission to the hospital for clean-out occurred for 13.8% of patients in the pre-lead exposure period and for 8% in the post-lead exposure period, with no statistically significant difference between the two groups. ED intervention for constipation was required in 24.1% of the pre-lead exposure group and only 4% of the post-lead exposure group. The difference was statistically significant, with a P value of 0.01. ED interventions include cleaning out and the use of an enema or suppository while in the ED.

Abdominal pain was the most common presentation in both groups, with 69% in the pre-lead exposure group and 62% in the post-lead exposure group. Straining was significantly noted, appearing in 60% of the post-lead exposure group, while it was noted in 34.5% of the pre-lead exposure group. The difference was statistically significant, with a P value of 0.029. There was no significant difference noted in blood in the stool in both groups, with 24.1% and 28% in the pre-lead exposure and post-lead exposure groups, respectively. Fecal incontinence showed similar results.

Abdominal distention was the least common in both groups, with 6.9% and 8% in the pre-lead exposure and post-lead exposure groups, respectively, with no statistical difference noted between both groups. Stool impaction was noted in 20.7% of the pre-lead exposure group and only 12% of the post-lead exposure group; however, the difference was not statistically significant. The rest of the results are summarized in Table 2.

**Table 2:** Results of data analysis and the difference between both groups in percentage UTI: urinary tract infection

	Pre-Lead exposure	Post-Lead exposure	P Value
Hospital admission	4 (13.8%)	4 (8%)	0.456
ED intervention	7 (24.1%)	2 (4%)	0.01
Straining	10 (34.5%)	30 (60%)	0.029
Blood in the stool	7 (24.1%)	14 (28%)	0.78
Fecal incontinence	6 (20.7%)	16 (32%)	0.28
Abdominal pain	20 (69%)	31 (62%)	0.53
Vomiting	7 (24.1%)	8 (16%)	0.374
Hx of UTI	2 (6.9%)	5 (10%)	0.6
Abdominal distention	2 (6.9%)	4 (8%)	0.85
Stool impaction	6 (20.7%)	6 (12%)	0.34

X-rays showed abnormal findings suggestive of constipation in 86% of the patients. The main treatment was polyethylene glycol in 89%-92% of the patients. The rest were dulcolax, senna, and milk of magnesia. Enemas were used for therapy in 24.1% of the pre-lead exposure group and only 4% of the post-exposure group, which mostly reflects more outpatient treatment options in 2017 compared to 2013 and more ED visits in 2013 compared to 2017. More than one line of treatment was used in 17.2% of the pre-lead exposure group and in 20% of the post-lead exposure group. Fourteen patients had a follow-up appointment, and from these, 10 patients reported improvement in the initial management plan, while in the post-lead exposure group, 36 patients had a follow-up appointment, and 19 patients reported improvement in the initial management plan. The difference was not statistically significant, with a P value of 0.23. The lead level was not found in 74% of the patients. Of the 23 patients who had a blood lead level, it was within the normal range.

## Discussion

Functional constipation is a common childhood disease with a prevalence of 10%. Many risk factors are linked to functional constipation, like a poor fiber diet, a sedentary lifestyle, poor toilet training, anxiety, and depression [1]. The pathophysiology behind functional constipation is unknown. Theories include anorectal dysfunctions with altered sensation, or colonic dysfunctions with altered motility and electrophysiology [11]. Lead poisoning can have a variety of effects on children. It causes fatigue, anemia, peripheral neuropathy, renal injury, and in severe cases coma and seizures. It might also be asymptomatic. From a gastrointestinal point of view, lead poisoning can cause abdominal pain and constipation. Around 20% of the patients who had constipation were found to have elevated lead blood levels in Tehran [12]. Gastrointestinal manifestations due to lead poisoning can be explained by lead’s inhibitory effect on the intestine's neural plexus and intestinal smooth muscle contraction [13].

The demography of our study sample reflects the city of Flint. In our study, we noted that the number of patients referred to GI clinics increased from 29 per year to 50 per year post-lead exposure. While we saw a difference in age between the time periods, we do not have a clear explanation for this. One explanation is that the younger age group is less exposed to lead, as mothers usually prefer to prepare the formula with processed water, while older children might drink more unprocessed water. Abdominal pain was the most common symptom associated with constipation in both groups. A similar finding was noted in another study, as constipation was the most frequent cause of acute abdominal pain visits in children [14].

Straining was significantly higher in the post-lead exposure group and was almost double that of pre-lead exposure. This finding could be linked to lead exposure. Our theory is that lead could affect intestine innervation, intestinal motility, or muscle function, especially since lead has been linked to causing neurological manifestations and muscle weakness in children [10]. However, further studies need to be done.

ED visits were more common in the 2013 group compared to the 2017 group, with fewer but not a statistically significant increase in admissions for clean-out. This likely reflects improved outpatient management of constipation over the years. There was no clinical or statistically significant difference noted in abdominal pain, blood in the stool, fecal incontinence, vomiting, history of urinary tract infection (UTI), abdominal distention, or stool impaction between both groups.

Imaging, including X-rays, was suggestive of constipation in 86% of the patients. This percentage is higher than what was documented in a previous study [15]. Of note, many of these X-rays were reported normal by radiologists, but after being reviewed by a gastroenterologist, they were noted to have an increased fecal load. An X-ray is not considered a reliable way to diagnose constipation [15]. Our study's findings do not suggest otherwise.

In both groups, the main treatment was osmotic laxatives, mostly polyethylene glycol. Any difference in therapies used in the pre-lead and post-lead exposure groups likely reflects the increase in outpatient options in the later time period. Using multiple treatments and follow-up appointments was similar in both time periods. The majority of patients report improvement, with no difference in percentage in both groups. This finding does not suggest the Flint water crisis increased the severity of constipation in children. Also, osmotic laxatives are still effective in treating constipation that might be due to lead poisoning. However, the latter will need further study to confirm.

The study was done in the Hurley Pediatric GI Clinic; Hurley Medical Center is considered the main hospital for children in Flint. Also, the pediatric gastroenterologist was the same in 2013 and 2017, which may mean fewer variations in assessment and management. On the other hand, our study has significant limitations. The number of patients in the study was low, which might affect the power of the study. Our patients were from one clinic, and by that, we might have missed a bulk of patients who presented with constipation and were managed by a primary care physician. Also, our study was done in one hospital, and there are other healthcare facilities for children besides Hurley Medical Center in Flint. Another limitation is that the lead level was not measured in most of our patients. We are looking into expanding our search to include more patients by adding extra time before and after exposure and collaborating with other clinics and medical centers in Flint to include their data in future studies.

## Conclusions

The number of patients referred to Hurley’s Pediatric GI Clinic for constipation increased after the lead water crisis in Flint. Moreover, straining has significantly increased in post-lead exposure compared to pre-lead exposure. There was no clinical or statistically significant difference noted in abdominal pain, blood in the stool, fecal incontinence, vomiting, history of UTI, abdominal distention, or stool impaction between both groups. A larger study would need to be done to confirm these findings, rule out other cofactors, and look into minerals in water and their effect on intestine innervations.
